# The Role of Immunotherapy in the Treatment of Gynecologic Cancers: A Systematic Review

**DOI:** 10.7759/cureus.65638

**Published:** 2024-07-29

**Authors:** Olugbenga Farajimakin

**Affiliations:** 1 Clinical Research, Faraj-Lab Medical Diagnostics and Research Centre, Lagos, NGA

**Keywords:** combination therapies, progression-free survival, immune checkpoint inhibitors, immunotherapy, gynecologic cancers

## Abstract

Gynecologic cancers remain a significant health burden worldwide. Immunotherapy has emerged as a promising treatment approach across various cancer types. To evaluate the efficacy and safety of immune checkpoint inhibitors, alone or in combination with other therapies, in the treatment of gynecologic cancers. We searched PubMed/MEDLINE, Cochrane Library, Embase, Web of Science, and ClinicalTrials.gov for clinical trials of immunotherapy in gynecologic cancers. Randomized controlled trials and prospective studies were included. MMAT tool was used to assess the quality of the studies. Risk of bias was assessed using appropriate tools for each study design. Seventeen studies met inclusion criteria, encompassing ovarian, endometrial, and cervical cancers. Immune checkpoint inhibitors, particularly in combination with standard therapies, demonstrated improved progression-free survival across multiple trials. Notable results include improved outcomes with pembrolizumab in endometrial and cervical cancers, and promising combinations of PARP inhibitors with checkpoint inhibitors in ovarian cancer. Safety profiles were generally consistent with known effects of immunotherapy. Immunotherapy shows significant promise in improving outcomes for patients with gynecologic cancers. Further research is needed to optimize patient selection and combination strategies.

## Introduction and background

Gynecologic cancers, including malignancies of the ovaries, uterus, cervix, vulva, and vagina, remain a significant challenge in oncology. These cancers contribute substantially to global morbidity and mortality, with ovarian cancer alone causing over 200,000 deaths annually [1,2]. Despite advancements in surgical techniques, chemotherapy regimens, and targeted therapies, many patients experience recurrence or disease progression, underscoring the urgent need for innovative treatment approaches [3]. The intricate relationship between cancer cells and the immune system has long been recognized as a crucial factor in tumor development and progression [4]. In recent years, immunotherapy has emerged as a groundbreaking treatment modality across various cancer types, offering new hope for patients with previously limited options [5]. Immune checkpoint inhibitors (ICIs) have shown remarkable efficacy in several solid tumors by reactivating the body's immune response against cancer cells [6].

ICIs primarily target the programmed cell death protein 1 (PD-1), its ligand (PD-L1), or the cytotoxic T-lymphocyte-associated protein 4 (CTLA-4) pathways [7]. By blocking these inhibitory signals, ICIs reinvigorate exhausted T-cells and promote anti-tumor immunity [8]. The success of ICIs in melanoma, non-small cell lung cancer, and other malignancies has prompted extensive investigation into their potential in gynecologic cancers [9,10]. Immunotherapy shows promise in various gynecologic cancers including ovarian cancer, often diagnosed at advanced stages, exhibits immunogenic properties that make it a promising candidate for immunotherapy [11]. The presence of tumor-infiltrating lymphocytes (TILs) in ovarian cancer correlates with improved prognosis, suggesting a role for immune-mediated tumor control [12]. Additionally, the high mutational burden observed in some ovarian cancer subtypes may contribute to neoantigen formation and enhanced immunogenicity [1].

Additionally, endometrial cancer, particularly the microsatellite instability-high (MSI-H) subtype, has demonstrated responsiveness to checkpoint inhibition [13]. The high mutational load and increased neoantigen burden in MSI-H tumors create a favorable environment for immunotherapy [14]. Moreover, the presence of TILs and PD-L1 expression in endometrial cancer has been associated with improved outcomes, suggesting potential benefits from immune checkpoint blockade [15]. Furthermore, in cervical cancer, where human papillomavirus (HPV) infection plays a crucial etiological role, immunotherapy offers a rational approach to enhance anti-tumor immune responses [16]. The expression of viral antigens by cervical cancer cells provides unique targets for immune recognition and attack [17]. Furthermore, the immunosuppressive microenvironment often observed in cervical cancer may be effectively modulated by checkpoint inhibitors [18].

Early clinical trials evaluating ICIs as monotherapy in gynecologic cancers showed modest activity, prompting an investigation into combination strategies [19,20]. The integration of immunotherapy with standard treatments such as chemotherapy, targeted agents (e.g., poly(ADP-ribose) polymerase [PARP] inhibitors), and radiotherapy has emerged as a promising approach to potentiate anti-tumor immunity and improve clinical outcomes [21,22]. Combining chemotherapy with immunotherapy may enhance tumor immunogenicity through increased neoantigen release and immunogenic cell death [23]. PARP inhibitors, which have shown significant efficacy in BRCA-mutated ovarian cancers, may synergize with checkpoint inhibitors by increasing mutational load and promoting T-cell infiltration [24]. Radiotherapy, known to modulate the tumor microenvironment and enhance antigen presentation, has also been explored in combination with immunotherapy to potentially induce abscopal effects [25].

Despite these promising developments, challenges remain in optimizing immunotherapy for gynecologic cancers. The identification of predictive biomarkers, such as PD-L1 expression, tumor mutational burden, and immune gene signatures, is crucial for patient selection and treatment personalization [26,27]. Additionally, understanding and managing immune-related adverse events is essential for the safe and effective implementation of these therapies [28]. The evolving landscape of immunotherapy in gynecologic cancers also includes the exploration of novel approaches such as adoptive cell therapies, cancer vaccines, and bispecific antibodies [29-31]. These strategies aim to further enhance the specificity and potency of anti-tumor immune responses, potentially offering new options for patients who do not respond to current immunotherapies.

This systematic review aims to evaluate the current evidence for the efficacy and safety of immunotherapy, focusing on ICIs, in gynecologic cancers. By synthesizing data from recent phases 2 and 3 clinical trials, we seek to provide a comprehensive overview of immunotherapy's role across ovarian, endometrial, and cervical cancers. Our analysis encompasses various treatment strategies, including ICI monotherapy, combinations with chemotherapy or targeted agents, and integration with radiotherapy. We also discuss ongoing trials, emerging biomarkers, and future directions in the field, to inform clinical decision-making and guide future research efforts in this rapidly evolving area of oncology [32-34].

The objectives of this review are to assess the efficacy of ICIs in terms of progression-free survival (PFS), overall survival (OS), and objective response rates (ORR) across different gynecologic cancer types. Furthermore, to evaluate the safety profile of immunotherapy in these populations, including the incidence and management of immune-related adverse events. Moreover, to identify potential biomarkers of response to immunotherapy in gynecologic cancers and examine the effectiveness of various combination strategies involving immunotherapy. Finally, to highlight current clinical indications and ongoing areas of research in the field.

By addressing these objectives, we aim to provide clinicians and researchers with a critical appraisal of the current state of immunotherapy in gynecologic cancers, informing clinical decision-making and guiding future research directions in this rapidly evolving field.

## Review

Research question and protocol

This systematic review aims to address the question: What is the current role of immunotherapy in the treatment of gynecologic cancers?" To structure our investigation, we employed the PICO (Population, Intervention, Comparison, Outcome) framework. The population (P) consists of patients with gynecologic cancers, including ovarian, cervical, endometrial, vulvar, and vaginal cancers. The intervention (I) focuses on immunotherapy, encompassing checkpoint inhibitors, CAR-T cell therapy, and cancer vaccines. For comparison (C), we consider standard treatments such as chemotherapy, radiation, and surgery, as well as placebo or no comparison in single-arm studies. Our outcomes (O) of interest include efficacy measures (overall survival, progression-free survival (PFS), objective response rate), safety (adverse events), biomarkers of response, and current clinical indications. This comprehensive approach allows us to explore not only the efficacy and safety of immunotherapy but also its current place in treatment algorithms, ongoing clinical trials, and potential future directions in gynecologic oncology. The details of PICO are described in Table 1.

**Table 1:** PICO framework. CAR-T, Chimeric Antigen Receptor T-cell; PICO, Population, Intervention, Comparison, Outcome

Component	Description
Population (P)	Patients with gynecologic cancers (ovarian, cervical, endometrial, vulvar, and vaginal)
Intervention (I)	Immunotherapy (e.g., checkpoint inhibitors, CAR-T-cell therapy, and cancer vaccines)
Comparison (C)	Standard treatment (e.g., chemotherapy, radiation, surgery), placebo, or no comparison (single-arm studies)
Outcomes (O)	Efficacy (overall survival, progression-free survival, and objective response rate), safety (adverse events), biomarkers of response, and current clinical indications

Eligibility criteria

We included randomized controlled trials and prospective studies evaluating immune checkpoint inhibitors in ovarian, endometrial, or cervical cancer. To be eligible, studies had to report on efficacy outcomes, such as PFS or overall survival (OS), and/or safety data. Our inclusion criteria specified that articles should be published in English within the last 10 years from (January 2014-June 2024), encompassing clinical trials (Phases I, II, and III) and observational studies focused on immunotherapy in gynecologic cancers. We excluded preclinical studies, case reports, studies not primarily focused on immunotherapy, and those not specific to gynecologic cancers to ensure the relevance and quality of our review.

Protocol

Our systematic review follows a rigorous protocol to ensure comprehensive and unbiased results. We begin with a thorough literature search across multiple databases to identify relevant studies. Following the initial search, we screen studies based on our predefined inclusion and exclusion criteria to select the most pertinent research. For studies meeting our criteria, we extract relevant data and assess the quality of each included study using appropriate evaluation tools. After data extraction and quality assessment, we synthesize the findings to identify patterns, trends, and key insights across the body of literature. To ensure transparency and reproducibility in our methodology and reporting, we adhere to the Preferred Reporting Items for Systematic Reviews and Meta-Analyses (PRISMA) guidelines throughout the review process.

Information sources and search strategy

We conducted a comprehensive search of PubMed/MEDLINE, Cochrane Library, Embase, Web of Science, and ClinicalTrials.gov from inception to January 2024. Our search strategy utilized a combination of terms related to gynecologic cancers and immunotherapy. For gynecologic cancers, we included terms such as "gynecologic cancer," "ovarian cancer," "cervical cancer," "endometrial cancer," "uterine cancer," "vulvar cancer," and "vaginal cancer." Immunotherapy-related terms encompassed "immunotherapy," "immune checkpoint inhibitor," "PD-1 inhibitor," "PD-L1 inhibitor," "CTLA-4 inhibitor," "CAR-T cell therapy," "cancer vaccine," and "adoptive cell therapy." We also included general terms like "randomized controlled trial" and "clinical trials" to focus on high-quality clinical research. These terms were combined using appropriate Boolean operators to optimize our search results. Table 2 provides details on MeSH terminologies used in the PICO framework.

**Table 2:** Corresponding MeSH terms. CTLA-4, cytotoxic T-lymphocyte-associated protein 4; PD-1, programmed cell death protein 1; PD-L1, programmed death-ligand 1

PICO component	MeSH terms
Population (P)	Genital neoplasms, genital cancer, female, ovarian neoplasms, ovarian cancer, uterine neoplasms, uterine cancer, endometrial neoplasms, endometrial cancer, cervical neoplasms, cervical cancer, vulvar neoplasms, vulvar cancer, vaginal neoplasms, vaginal cancer
Intervention (I)	Immunotherapy, adoptive immunotherapy, cancer vaccines, checkpoint inhibitor therapy, programmed cell death 1 receptor, CTLA-4 antigen, immune checkpoint inhibitors, PD-1 inhibitors, PD-L1 inhibitors, CTLA-4 inhibitors
Comparison (C)	Clinical trials as topic, randomized controlled trials as topic, clinical trials, phase I as topic, clinical trials, phase II as topic, clinical trials, phase III as topic
Outcomes (O)	Treatment outcome, survival analysis, disease-free survival, progression-free survival, adverse effects, biomarkers, tumor

Study selection process

**Figure 1:**
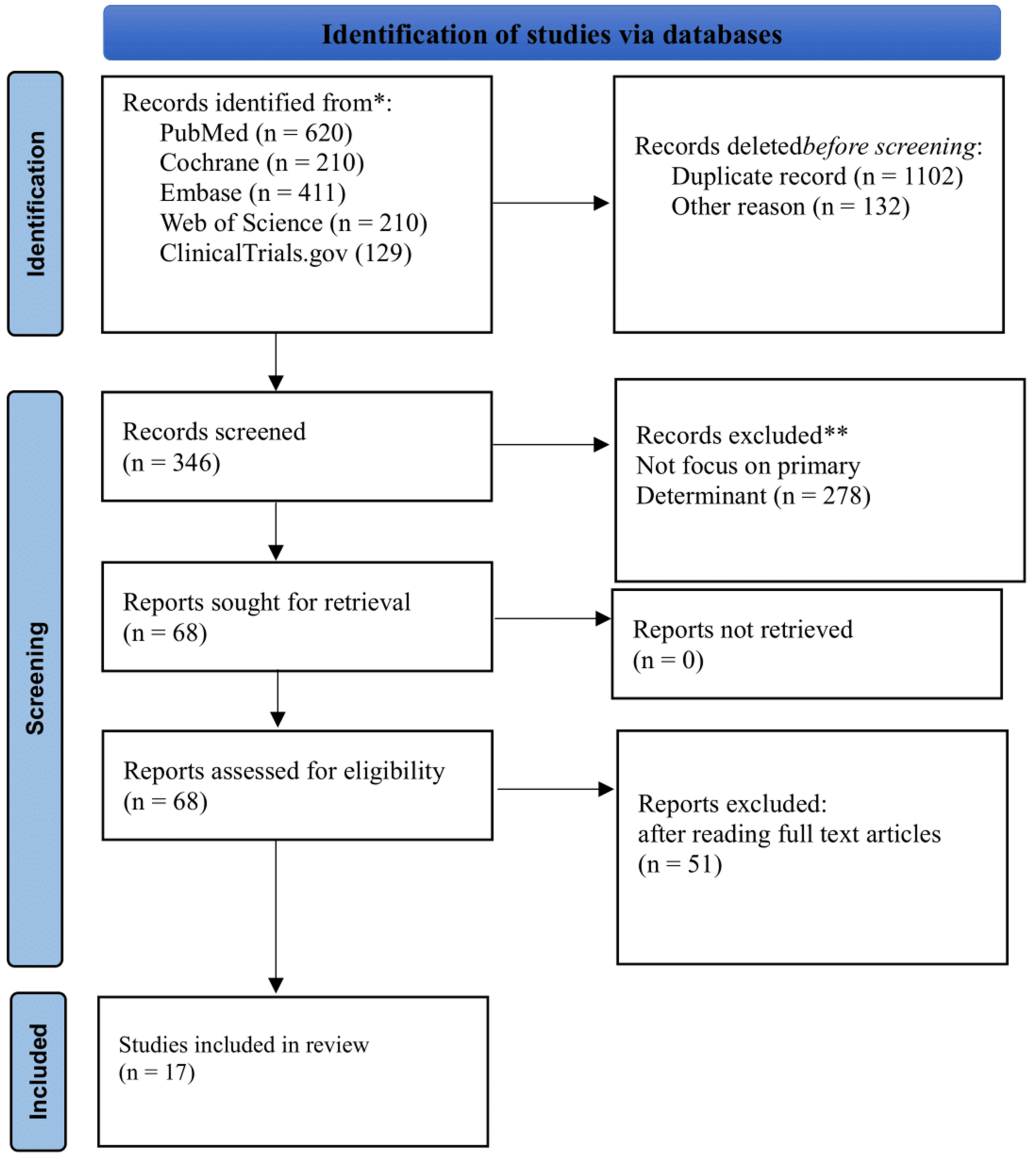
PRISMA flowchart for selection of study. PRISMA, Preferred Reporting Items for Systematic Reviews and Meta-Analyses

Our study selection process involved two independent reviewers who screened titles and abstracts, followed by a full-text review of potentially relevant articles. Any disagreements were resolved through consensus or by consulting a third reviewer. This approach ensured a systematic and unbiased selection of relevant studies for our review. Our initial search identified a total of 1,580 records across the databases: PubMed/MEDLINE (620), Cochrane Library (210), Embase (411), Web of Science (210), and ClinicalTrials.gov (129). The selection process followed the PRISMA (Preferred Reporting Items for Systematic Reviews and Meta-Analyses) flow guidelines, as illustrated in Figure 1 (PRISMA flowchart for study selection).

Data collection and risk-of-bias assessment

For data collection, two reviewers independently extracted information from the selected studies using a standardized form. This process helped ensure the accuracy and completeness of the extracted data. To assess the risk of bias in our included studies, we employed the Mixed-Methods Appraisal Tool (MMAT). This tool enables a comprehensive evaluation of potential biases affecting study validity, thereby enhancing the reliability of our systematic review findings.

MMAT is utilized to assess the methodological quality of various study types, including qualitative, quantitative, and mixed-methods research. When interpreting results using the MMAT, researchers evaluate studies based on five criteria specific to each study category. Each criterion is rated as *Yes*, *No*, or *Can't tell*. Unlike some other quality assessment tools, the MMAT does not produce an overall numerical score. Instead, it provides a more nuanced, criterion-by-criterion assessment of a study's methodological rigor. The quality of a study is generally considered higher the more *Yes* responses it receives, while *No* or *Can't tell* responses indicate potential weaknesses or lack of clarity in reporting. Results are typically reported by stating the number of *Yes* responses out of the total applicable criteria, often accompanied by a descriptive analysis of the identified strengths and weaknesses. The MMAT does not offer a specific cutoff score for determining *good* or *poor* quality; rather, it encourages a more detailed interpretation of methodological quality. In systematic reviews, MMAT results can be used to compare quality across studies and inform the synthesis of evidence by indicating how much weight or confidence should be given to different studies' findings. It's important to note that the MMAT assesses methodological quality, not the overall importance or impact of a study's results. For transparency, full MMAT assessment results for each study are often provided in supplementary materials. This approach to quality assessment allows for a comprehensive understanding of each study's methodological strengths and limitations within the context of the research question being addressed.

Results

The systematic review included 17 studies, comprising 11 phase 3 trials, 3 phase 2 trials, 2 phase 1/2 trials, and 1 review article. The studies were distributed across different gynecologic cancer types, with 8 studies focusing on ovarian cancer, 3 on endometrial cancer, 5 on cervical cancer, and 1 study encompassing multiple gynecologic cancer types. Table 3 provides detailed functional characteristics of these studies.

**Table 3:** Explains the functional details of the studies. OS, overall survival; ORR, objective response rate; MTB, maximum tolerated dose; dMMR, mismatch repair deficiency; HRD, homologous recombination deficiency; ISD, individualized starting dose; MSI-H, microsatellite instability-high

Sr. No	Author	Drug	Cancer type	Phase	Sample size	Primary endpoint	Key results
1	Mirza et al., 2016 (ENGOT-OV16/NOVA trial) [35]	Niraparib	Platinum-sensitive recurrent ovarian cancer	3	553	Progression-free survival (PFS)	gBRCA cohort: 21.0 vs. 5.5 months (HR 0.27) non-gBRCA HRD cohort: 12.9 vs. 3.8 months (HR 0.38); overall non-gBRCA cohort: 9.3 vs. 3.9 months (HR 0.45)
2	Moore et al., 2019 [36]	Ipafricept + paclitaxel/carboplatin	Recurrent platinum-sensitive ovarian cancer	1b	37	Maximum tolerated dose	ORR: 75.7%; median PFS: 10.3 months; median OS: 33 months
3	Konstantinopoulos et al., 2019 (TOPACIO/KEYNOTE-162) [21]	Niraparib + pembrolizumab	Recurrent ovarian carcinoma	1/2	62 (ovarian cohort)	Objective response rate	ORR: 18% (5% CR, 13% PR); disease control rate: 65%
4	Haddley, 2019 [37]	Dostarlimab	Advanced solid tumors (focus on NSCLC and endometrial cancer)	Overview of phases I-III studies	Not specified	Varies	Preliminary data showed durable partial responses in select patients with NSCLC and endometrial cancer
5	Moore et al., 2019 (IMagyn050/GOG 3015/ENGOT-OV39 trial) [38]	Atezolizumab + paclitaxel/carboplatin/bevacizumab	Newly diagnosed stage III or IV ovarian, fallopian tube, or primary peritoneal cancer	3	1300 (planned)	PFS and overall survival	Improved PFS and OS
6	Oaknin et al., 2020 (GARNET trial) [39]	Dostarlimab	dMMR endometrial cancer	1	71	Objective response rate	ORR: 42.3% (12.7% CR, 29.6% PR); median duration of response not reached
7	González-Martin et al., 2021 (KEYNOTE-826) [40]	Pembrolizumab + chemotherapy +/- bevacizumab	Persistent, recurrent, or metastatic cervical cancer	3	617	PFS and overall survival	Median PFS: 10.4 vs. 8.2 months (HR 0.65) 24-month OS: 50.4% vs 40.4% (HR 0.67)
8	González-Martin et al., 2021 (ENGOT-Ov41/GEICO 69-O/ANITA trial) [40]	Atezolizumab + niraparib + platinum-based chemotherapy	Recurrent ovarian, tubal, or peritoneal cancer with platinum-free interval >6 months	3	414 (planned)	PFS	Improved PFS
9	Monk et al., 2021 (ATHENA trial) [41]	Rucaparib +/- nivolumab	Newly diagnosed ovarian cancer	3	1000	PFS	Improved PFS
10	Monk et al., 2022 (ATHENA-MONO trial) [42]	Rucaparib	Newly diagnosed ovarian cancer	3	538	PFS	HRD population: Median PFS 28.7 vs. 11.3 months (HR 0.47, *P *= 0.0004) ITT population: median PFS 20.2 vs. 9.2 months (HR 0.52, *P *< 0.0001) HRD-negative population: median PFS 12.1 vs. 9.1 months (HR 0.65)
11	Tewari et al., 2022 (EMPOWER-Cervical 1/GOG-3016/ENGOT-cx9 trial) [43]	Cemiplimab	Recurrent cervical cancer	3	608	Overall survival	Median OS: 12.0 vs. 8.5 months (HR 0.69) Median PFS: HR 0.75, ORR: 16.4% vs. 6.3%
12	Mirza et al., 2023 (PRIMA/ENGOT-OV26/GOG-3012 trial) [44]	Niraparib (individualized starting dose)	Newly diagnosed advanced ovarian cancer	3	733	PFS	Similar efficacy with improved safety for individualized vs. fixed starting dose; overall HR: 0.69 (ISD) vs. 0.59 (FSD); HRD population HR: 0.39 (ISD) vs. 0.44 (FSD)
13	Monk et al., 2023 (CALLA trial) [45]	Durvalumab + chemoradiotherapy	Locally advanced cervical cancer	3	770	PFS	Median PFS not reached in either group (HR 0.84, *P *= 0.17); 12-month PFS: 76.0% vs. 73.3%
14	Kurtz et al., 2023 (ATALANTE/ENGOT-ov29 trial) [46]	Atezolizumab + bevacizumab + chemotherapy	Platinum-sensitive ovarian cancer	3	614	PFS	ITT population: Median PFS 13.5 vs. 11.3 months (HR 0.83, *P *= 0.041); PD-L1+ population: Median PFS 15.2 vs. 13.1 months (HR 0.86, *P* = 0.30)
15	Pignata et al., 2023 (MITO END-3 trial) [47]	Avelumab + carboplatin/paclitaxel	Advanced or recurrent endometrial cancer	2	125	PFS	Median PFS: 9.6 vs. 9.9 months (HR 0.78, *P *= 0.085)
16	Eskander et al., 2023 (NRG-GY018 trial) [48]	Pembrolizumab + paclitaxel/carboplatin	Advanced or recurrent endometrial cancer	3	816	PFS	dMMR cohort: 12-month PFS 74% vs. 38% (HR 0.30); pMMR cohort: median PFS 13.1 vs. 8.7 months (HR 0.54)
17	Lorusso et al. 2024 (ENGOT-cx11/GOG-3047/KEYNOTE-A18 trial) [49]	Pembrolizumab + chemoradiotherapy	Newly diagnosed, high-risk, locally advanced cervical cancer	3	1060	PFS and overall survival	24-month PFS: 68% vs. 57% (HR 0.70, *P *= 0.0020); 24-month OS: 87% vs. 81% (HR 0.73, not statistically significant)

Synthesis of results

Mechanism of Action

Immunotherapy, particularly checkpoint inhibitors, works by blocking proteins that prevent T-cells from attacking cancer cells. The most common targets are PD-1, programmed death-ligand 1 (PD-L1), and CTLA-4. By inhibiting these checkpoints, the immune system's ability to recognize and destroy cancer cells is enhanced.

Efficacy in different gynecologic cancers

Ovarian Cancer

Niraparib maintenance therapy showed significant PFS improvement in platinum-sensitive recurrent ovarian cancer [35]. Combination of niraparib with pembrolizumab showed promise in recurrent ovarian cancer [21]. Rucaparib maintenance demonstrated PFS benefit in newly diagnosed ovarian cancer [42]. Atezolizumab added to bevacizumab and chemotherapy did not significantly improve PFS in platinum-sensitive ovarian cancer [46].

Endometrial Cancer

Dostarlimab showed durable antitumor activity in mismatch repair-deficient (dMMR) endometrial cancer [39]. Avelumab added to carboplatin and paclitaxel showed potential benefit in advanced or recurrent endometrial cancer [47]. Pembrolizumab plus chemotherapy significantly improved PFS in advanced endometrial cancer [48].

Cervical Cancer

Pembrolizumab improved PFS and OS when added to chemotherapy in recurrent or metastatic cervical cancer [50]. Cemiplimab demonstrated survival benefit in recurrent cervical cancer [43]. Durvalumab did not significantly improve PFS when added to chemoradiotherapy in locally advanced cervical cancer [45]. Pembrolizumab plus chemoradiotherapy improved PFS in newly diagnosed, high-risk, locally advanced cervical cancer [49].

Biomarkers and patient selection

PD-L1 expression has been used as a biomarker in some trials, but its predictive value varies across studies and cancer types. Mismatch repair deficiency (dMMR) or microsatellite instability-high (MSI-H) status appears to be a strong predictor of response to immunotherapy, particularly in endometrial cancer. Homologous recombination deficiency (HRD) status may also influence response, especially when combining immunotherapy with PARP inhibitors.

Combination strategies

Immunotherapy + Chemotherapy

This combination has shown promise in cervical and endometrial cancers.

Immunotherapy + PARP Inhibitors

This approach is being extensively studied in ovarian cancer, with potential synergistic effects.

Immunotherapy + Antiangiogenic Agents

Combinations with bevacizumab have shown efficacy in cervical cancer.

Immunotherapy + Radiotherapy

The addition of immunotherapy to chemoradiotherapy is being explored in locally advanced cervical cancer.

Safety profile

Immune-related adverse events (irAEs) are a significant concern with immunotherapy. Across the studies, the safety profiles of immunotherapies and PARP inhibitors were generally consistent with previous reports. Common adverse events included fatigue, nausea, and anemia for PARP inhibitors, and irAEs for checkpoint inhibitors. The individualized dosing approach for niraparib in the PRIMA trial demonstrated improved tolerability without compromising efficacy [21,35-49].

Discussion

The landscape of gynecologic cancer treatment has undergone a significant transformation with the advent of targeted therapies and immunotherapy. PARP inhibitors have emerged as a cornerstone in the management of ovarian cancer, particularly for patients with BRCA mutations or HRD. The PRIMA trial by González-Martin et al. demonstrated the efficacy of niraparib as first-line maintenance therapy, showing significant improvements in PFS across all biomarker subgroups [32]. This finding was corroborated by the SOLO-1 trial, where Moore et al. showed significant PFS improvement with olaparib maintenance in newly diagnosed advanced ovarian cancer with BRCA mutations [36]. Furthermore, Coleman et al. in the VELIA/GOG-3005 study evaluated veliparib added to first-line chemotherapy and continued as maintenance, showing improved PFS in HRD-positive patients [51]. These studies collectively support the role of PARP inhibitors in ovarian cancer treatment, especially in patients with specific genetic profiles.

Immunotherapy has shown promise across various gynecologic cancers. In cervical cancer, the KEYNOTE-826 trial by Colombo et al. reported significant improvements in PFS and OS with the addition of pembrolizumab to chemotherapy in the first-line setting [50]. This finding is complemented by the CheckMate 358 trial, where Naumann et al. evaluated nivolumab in recurrent/metastatic cervical cancer, showing promising activity [52]. Tewari et al. in the EMPOWER-Cervical 1/GOG-3016/ENGOT-cx9 study demonstrated improved OS with cemiplimab compared to chemotherapy in recurrent cervical cancer [43]. For endometrial cancer, Oaknin et al. demonstrated remarkable activity of dostarlimab in dMMR tumors in a phase 1 trial [39], supported by the KEYNOTE-158 trial, where O'Malley et al. showed durable responses with pembrolizumab in previously treated, advanced endometrial cancer, particularly in MSI-H/dMMR tumors [42].

However, the integration of immunotherapy into treatment strategies has yielded mixed results in some trials. Monk et al. reported that the CALLA study, investigating durvalumab in locally advanced cervical cancer, did not meet its primary endpoint [45]. Similarly, Kurtz et al. found no significant improvement in PFS with atezolizumab in platinum-sensitive ovarian cancer in the ATALANTE trial [46]. These results underscore the need for refined patient selection strategies and more robust predictive biomarkers.

The combination of PARP inhibitors and immunotherapy represents an intriguing approach. Konstantinopoulos et al. reported encouraging activity with niraparib plus pembrolizumab in platinum-resistant ovarian cancer in the TOPACIO/KEYNOTE-162 trial, particularly in BRCA-mutated tumors [21]. This strategy is further supported by the MEDIOLA trial, where Drew et al. evaluated the combination of olaparib and durvalumab in germline BRCA-mutated platinum-sensitive ovarian cancer, showing promising activity [53]. The ongoing ATHENA trial, described by Monk et al., evaluating rucaparib with or without nivolumab as first-line maintenance therapy in ovarian cancer, may provide valuable insights into the potential synergy between PARP inhibition and immunotherapy [41].

Recent advances in molecular profiling have led to a more nuanced understanding of gynecologic cancers. The Cancer Genome Atlas Research Network's comprehensive genomic analysis of ovarian carcinoma has provided valuable insights into molecular subtypes and potential therapeutic targets [54]. Building on this foundation, Massard et al. in the MOSCATO 01 trial demonstrated the feasibility and potential benefit of molecular profiling-guided therapy across various cancer types, including gynecologic cancers [55]. The ongoing NCI-MATCH trial described by Salama et al. is evaluating targeted therapies based on specific molecular alterations across multiple tumor types, including gynecologic cancers [56]. These studies underscore the growing importance of molecular profiling in guiding treatment decisions and support the trend toward more personalized treatment approaches.

The role of combination therapies is also evolving. Liu et al. investigated the combination of nivolumab and bevacizumab in relapsed ovarian cancer, demonstrating promising activity and highlighting the potential of combining immunotherapy with anti-angiogenic agents [22]. In endometrial cancer, Makker et al. reported encouraging results with the combination of lenvatinib and pembrolizumab in advanced diseases [33]. This finding is further supported by the LEAP-005 study, where Zhou et al. investigated lenvatinib plus pembrolizumab in previously treated advanced solid tumors, including gallbladder cancer, demonstrating encouraging results [57].

In conclusion, while significant strides have been made in the treatment of gynecologic cancers with targeted therapies and immunotherapy, many questions remain. Optimal patient selection, ideal combination strategies, and methods to overcome treatment resistance are areas that require further investigation. The integration of molecular profiling into clinical decision-making, as demonstrated by studies like MOSCATO 01 and NCI-MATCH, represents a promising avenue for personalized treatment approaches.

Ongoing research

Several large-scale phase III trials are ongoing to further define the role of immunotherapy in various gynecologic cancers and treatment settings. Research is focusing on identifying predictive biomarkers beyond PD-L1 expression to better select patients who are likely to benefit from immunotherapy. Novel combination strategies and sequencing of treatments are being explored to optimize outcomes.

Limitations

This systematic review, while comprehensive, has several notable limitations. The heterogeneity of included studies, covering various gynecologic cancers and treatment approaches, may complicate direct comparisons. Many studies are recent trials, potentially lacking long-term survival and safety data. The inconsistent predictive value of biomarkers across studies and cancer types poses challenges for establishing uniform patient selection criteria. The mixed results observed, with some trials showing significant benefits while others failed to meet primary endpoints, underscore the complexity of treatment responses in different patient populations. The review may be subject to publication bias, favoring positive results. The scarcity of head-to-head comparisons between different immunotherapy agents or combination strategies hinders the determination of optimal treatment approaches. Given the rapidly evolving nature of immunotherapy and targeted therapies, some included studies may become outdated quickly. The focus on more common gynecologic cancers might overlook rarer subtypes. Variability in adverse event reporting across trials complicates safety profile comparisons. The review lacks cost-effectiveness analysis, an important factor for clinical implementation, and has limited discussion on quality-of-life outcomes. Finally, the potential for overlapping patient populations across trials, particularly in rare cancers or specialized treatment centers, could skew results. Addressing these limitations in future research and reviews would provide a more comprehensive understanding of immunotherapy and targeted therapies in gynecologic cancers.

Future recommendations

Future research should focus on identifying predictive biomarkers for patient selection, optimizing combination strategies, and exploring novel immunotherapy approaches. Long-term follow-up data from these trials will be crucial to understand the durability of responses and long-term safety profiles. Additionally, quality-of-life assessments and health economic analyses will be important to fully evaluate the impact of these new therapies on patient care and healthcare systems. Continued research and carefully designed clinical trials are necessary to address these important questions and further improve outcomes for patients with gynecologic cancers.

## Conclusions

Immune checkpoint inhibitors have demonstrated significant efficacy in improving outcomes for patients with gynecologic cancers, particularly when combined with standard therapies. The most robust evidence supports their use in endometrial and cervical cancers, while further research is needed to optimize their role in ovarian cancer. As the field evolves, ongoing and future studies will help clarify the optimal use of immunotherapy in clinical practice for gynecologic cancers.
